# Self-efficacy among pregnant and breastfeeding women with rheumatic and musculoskeletal diseases: an international cross-sectional pilot study

**DOI:** 10.1093/rap/rkag132

**Published:** 2026-10-24

**Authors:** Eugenia Y Chock, Lekshmi M Rahulan, Suhana Hussain, Ishaan Shinde, Hannah Maher, Vincenzo Venerito, Vahed Maroufy, Laura Andreoli, Ioannis Parodis, Samuel K Shinjo, Carlo V Caballero-Uribe, Dzifa Dey, Cristina Alexandru, Anca Bobircă, Claudia Cobilinschi, Karen Schreiber, Silvia Aguilera, COVAD Study Group, Chafia Dahou Makhloufi, Leandro Gabriel Ferreyra Garrott, Valentina Vardanyan, Vidya Sadanand Limaye, Jessica Day, A T M Tanveer Hasan, Marharyta Volkava, Sonam Yangchen, Dimitri Luz Felipe da Silva, Odirlei Andre Monticielo, Samuel Katsuyuki Shinjo, Simone Appenzeller, Russka Shumnalieva, Rositsa Svetoslavova Dacheva, Tsvetelina Velikova, Koy Rattanak, Fernando Kemta Lekpa, Océane Landon-Cardinal, Vanessa Ocampo, María Francisca Bozán Pérez, Antonia Valenzuela, Guochun Wang, Ho So, Iris Tang, Edmund K M Wong, Carlos Enrique Toro Gutiérrez, Sebastian Herrera Uribe, Carlo Vinicio Caballero-Uribe, Ivan Padjen, Zeljka Kardum, Konstantinos Parperis, Karen Schreiber, Jossiell Then Báez, Reem Hamdy Abdellatif Mohammed, Hala Mohamed Lotfy, Walaa Abdel Rahman Saleh, Mervat Eissa, Tamer A Gheita, Carlos Rios, Sandra Meisalu, Becky Abdissa Adugna, Margherita Giannini, Julien Campagne, Alain Meyer, Francis Guillemin, Hannah Labinsky, Gerd-Rüdiger Burmester, Johannes Knitza, Dzifa Dey, Alexandros Panagiotopoulos, Eirini Vasarmidi, Katerina Antoniou, Andrea Bran Ordóñez, Melinda Nagy-Vincze, Vikas Agarwal, Bhupen Barman, Yogesh Preet Singh, Arunkumar R Pande, Jasmine Parihar, Lekshmi M R , Meera Shah, Suryo Anggoro Kusumo Wibowo, Maryam Masoumi, Avin Maroof, Asal Adnan Redha, Marta Mosca, Nicoletta Del Papa, Fabiola Atzeni, Marco Sebastiani, Franco Franceschini, Pier Paolo Sainaghi, Maria Giovanna Danielli, Vincenzo Venerito, Alessandro Giollo, Laura Andreoli, Alessia Alluno, Devis Benfaremo, Valentino Paci, Giovanni Damiani, Elvis Hysa, Matteo Lucchini, Alberto Lo Gullo, Lorenzo Cavagna, Marco Fornaro, Akira Yoshida, Saori Abe, Masataka Kuwana, Ran Nakashima (Sasai), Fatima Alnaimat, Gulzhan Trimova, Dana Bekaryssova, Akerke Auanassova, Eugene Kalman Genga, Nelly Ziade, Soad Salem Hashad, Laila Ayoub, Syahrul Sazliyana Shaharir, Tan Chou Luan, Rosalie Magro, Erick Adrian Zamora Tehozol, Ignacio García-De La Torre, Iris J Colunga‑ Pedraza, Javier Merayo-Chalico, Deshire Alpizar-Rodriguez, Miguel Angel Saavedra Salinas, Abraham Edgar Gracia-Ramos, Victoria Sadovici-Bobeica, Zulgerel Dandii, Ihsane Hmamouchi, Binit Vaidya, Luis Fernando Perez Garcia, Nicola Dalbeth, Idania Calixta Escalante Mendoza, Christian Mauriel Uriarte Hernandez, Moussa Elh Ibrahim Fanata, Uyiekpen Ima-Edomwonyi, Ibukunoluwa Dedeke, Airenakho Emorinken, Henry Madu Nwankwo, Hakeem Babatunde Olaosebikan, Celestine Okwara, Tralagba Uchechukwu, Batool Hassan, Uzma Rasheed, Babur Salim, Nelly Colman Mc Leod, Manuel Francisco Ugarte-Gil, Victor Pimentel-Quiroz, Lisa S Traboco, Joanna Makowska, Aleksandra Opinc-Rosiak, Marcin Milchert, Luís Sousa Inês, Eduardo Dourado, Samar Al Emadi, Cristina Alexandru, Anca Bobircă, Claudia Cobilinschi, Margarita Aleksandrovna Gromova, Mubirigi Alexandre, Lina El Kibbe, Hussein Mohammed Halabi, Rada Miskovic, Anindita Santosa, Tze Chin Tan, Desmond Chua, Cristiana Sieiro Santos, Jesús Loarce-Martos, Sergio Prieto-González, Juan Molina-Collada, Chathurika Lakmini Dandeniya, Ahmed Seri Ibrahim Mohamed, Manasik Mamoun, Ioannis Parodis, Raphael Micheroli, Lisa Christ, Oliver Distler, Nada Alchama, Yi-Ming Chen, Chih-Wei Tseng, Phonpen Akarawatcharangura, Wanruchada Katchamart, Kotchakorn Kaewboot, Pichaya O-charoen, Wafa Hamdi, Döndü Üsküdar Cansu, Reşit Yıldırım, Tugba Izci Duran, Neslihan Gokcen, Myroslava Kulyk, Nesreen Mohamed Ismail Moustafa, Latika Gupta, Elena Nikiphorou, John D Pauling, Chris Wincup, Ai Lyn Tan, Tania Gudu, Valentina Pucino, Sarah Dyball, James Lilleker, Aurelie Najm, Arvind Nune, Yusuf A Rajabally, Anne-Marie Russell, Aarat Patel, Shikha Singla, Tulika Chatterjee, Ashima Makol, Mazen Dimachkie, Vahed Maroufy, Yurilís Fuentes-Silva, Bich Ngoc Nguyen, Lan Nguyen, Hung Nguyen, Thu Hoai Pham, Panganani Njobvu, Hugo Alonzo, Carlos Benito Santiago Pastelin, Generoso Guerra Bautista, Enrique Julio Giraldo Ho, Vikas Agarwal, Latika Gupta

**Affiliations:** Section of Rheumatology, Allergy, & Immunology, Department of Internal Medicine, Yale School of Medicine, New Haven, CT, USA; Department of Clinical Immunology and Rheumatology, Sanjay Gandhi Postgraduate Institute of Medical Sciences, Lucknow, India; Mahatma Gandhi Medical College and Hospital, Jaipur, India; Seth GS Medical College and KEM Hospital, Mumbai, India; University of Minnesota Medical School, Minneapolis, MN, USA; Department of Precision and Regenerative Medicine and Ionian Area, Rheumatology Unit, University of Bari, Bari, Italy; Department of Biostatistics and Data Science, School of Public Health, The University of Texas Health Center at Houston, Austin, TX, USA; Rheumatology and Clinical Immunology Unit, ASST Spedali Civili and University of Brescia, Brescia, Italy; Department of Clinical and Experimental Sciences, University of Brescia, Brescia, Italy; Danish Centre for Expertise in Rheumatology (CeViG), Danish Hospital for Rheumatic Diseases, Sønderborg, Denmark; Department of Regional Health Research, University of Southern Denmark, Odense, Denmark; Division of Rheumatology, Department of Medicine Solna, Karolinska Institutet, Karolinska University Hospital, and Center for Molecular Medicine (CMM), Stockholm, Sweden; Department of Rheumatology, Faculty of Medicine and Health, Örebro University, Örebro, Sweden; Division of Rheumatology, Faculdade de Medicina FMUSP, Universidade de São Paulo, São Paulo, SP, Brazil; Universidad de la Costa (CUC), Barranquilla, Colombia; Rheumatology Unit, Department of Medicine and Therapeutics, University of Ghana Medical School, College of Health Sciences, Korle-Bu, Accra, Ghana; Internal Medicine and Rheumatology Department, Dr. Ion Cantacuzino Clinical Hospital, Bucharest, Romania; Internal Medicine and Rheumatology Department, Dr. Ion Cantacuzino Clinical Hospital, Bucharest, Romania; Department of Rheumatology and Internal Medicine, Carol Davila University of Medicine and Pharmacy, Bucharest, Romania; Department of Rheumatology and Internal Medicine, Carol Davila University of Medicine and Pharmacy, Bucharest, Romania; Department of Rheumatology, Sfânta Maria Clinical Hospital, Bucharest, Romania; Danish Centre for Expertise in Rheumatology (CeViG), Danish Hospital for Rheumatic Diseases, Sønderborg, Denmark; Department of Regional Health Research, University of Southern Denmark, Odense, Denmark; Thrombosis and Haemophilia, Kings Healthcare Partners, Guy’s and St Thomas’ NHS Foundation Trust, London, UK; SAF Espana, ePAG at ERN-ReCONNET, patient representative within the EULAR Study Group on Reproductive Health and Family Planning (ReHFaP), Elche, Spain; Department of Clinical Immunology and Rheumatology, Sanjay Gandhi Postgraduate Institute of Medical Sciences, Lucknow, India; Department of Rheumatology, Royal Wolverhampton Hospitals NHS Trust, Wolverhampton, UK; Department of Inflammation and Ageing, School of Infection, Inflammation and Immunology, University of Birmingham, Birmingham, UK; Francis Crick Institute, London, UK

Key message

- Higher self-efficacy may support improved well-being during pregnancy in women with rheumatic and musculoskeletal diseases.

Dear Editor, Rheumatic and musculoskeletal diseases (RMDs) disproportionately affect women [1]. Although RA incidence rises with age, into and beyond menopause [2], many women are diagnosed during their childbearing years [3]. For these women, pregnancy and breastfeeding (PB) are physiologically and psychologically complex periods that may alter disease activity, symptom perception, treatment decisions and psychosocial well-being; clinical management during PB therefore requires careful balancing of maternal disease control and fetal safety [4]. Women living with RMDs during these stages often face challenges, including pain, fatigue and functional limitations, all while balancing their own well-being with the health of their children.

Self-efficacy (SE), an individual’s confidence in managing health-related demands, is an important factor in chronic disease populations, with higher SE associated with better pain management, function, psychological health and quality of life [5]. Because most people with RMDs are women of childbearing age, a knowledge gap remains as to whether SE is associated with better outcomes and lower disease activity in this group during PB. The bidirectional relationship between SE and patient-reported outcomes (PROs), including pain, fatigue, physical function and global health, remains insufficiently explored in women of reproductive age. Psychosocial constructs such as loneliness, resilience and perceived family support may also play a role in this association.

Using data from the multinational ‘Collating the Voice of People with Autoimmune Diseases-3’ international patient-reported e-survey, this study examined differences in SE among women with RMDs who were pregnant, breastfeeding or neither pregnant nor breastfeeding (Non-PB). Participants aged ≥18 years completed the survey, a 125-item instrument spanning 13 domains, including disease activity, multimorbidity [6], mental health, social determinants of health and pregnancy/lactation, alongside outcomes assessed using the Patient-Reported Outcomes Measurement Information System (PROMIS) [7]. Data were collected from February 2024 to October 2025. We examined demographic, obstetric, disease-related, psychological and social factors associated with SE among PB women with RMDs. Survey participants completed the Self-Efficacy for Managing Chronic Disease (SEMCD) scale, a six-item instrument that assesses an individual’s SE in managing physical discomfort, fatigue, pain, emotional distress and other symptoms that interfere with their goals while living with a chronic disease [8]. It also assesses participants’ confidence in reducing their need to see a doctor by undertaking certain tasks or activities on their own, and in doing things beyond taking medication to lessen the impact of illness on everyday life. The scale score was computed as the mean of the six items, and participants’ mean scores were then classified as low (≤33rd percentile), moderate (34th–66th percentile) or high (≥67th percentile) SE. Ethical approval was obtained from the Institutional Ethics Committee of the Sanjay Gandhi Postgraduate Institute of Medical Sciences (SGPGIMS), Lucknow, India (2023–209-EMP-133) and from the Institutional Review Boards of the participating countries where required. Informed consent was obtained electronically from all participants before they began the online survey.

A total of 23 pregnant and 52 breastfeeding women with RMDs participated in the survey, together with 1854 women with RMDs who were Non-PB. The PB group was younger (median age 34.0 years [pregnant] and 35.0 years [breastfeeding] vs 41.0 years for Non-PB) and more likely to be married or cohabiting (>90% vs 63.9%). More than two-thirds of pregnant women reported living comfortably on their present income, compared with 38.5% of breastfeeding and 37.2% of Non-PB women. Fewer than 10% of pregnant women with RMDs had active disease (8.7%), compared with breastfeeding women (34.6%) and Non-PB women (42.1%). Pregnant women reported lower pain severity (median visual analogue scale [VAS] 2.0 vs 4.0 and 5.0), lower fatigue (median VAS 5.0 vs 6.0 and 6.0), better physical function (PROMIS PF-4a T score 57.0 vs 46.9 and 41.9), better global physical health (PROMIS GPH 47.7 vs 39.8 and 37.4) and lower loneliness scores compared with breastfeeding and Non-PB women with RMDs (Table 1). None of the pregnant women with RMDs were taking high-dose corticosteroids, compared with 11.4% of the Non-PB group. Pregnant women recorded the highest median SEMCD score (7.00, interquartile range [IQR] 5.83–8.00), followed by breastfeeding women (5.92, IQR 4.50–7.37) and Non-PB women (5.50, IQR 4.17–7.00). Overall, 61% of pregnant women with RMDs were classified as having high SE, compared with 37% of breastfeeding and 32% of Non-PB women.

**Table 1:** Baseline characteristics of the study population by pregnancy/breastfeeding (PB) status.

Variable	RMD		
	Pregnant (*n* = 23)	Breastfeeding (*n* = 52)	Non-PB (*n* = 1854)
A. Demographic characteristics			
Age, years, median (IQR)	34.0 (31.0–36.5)	35.0 (31.0–39.0)	41.0 (33.0–46.0)
Caucasian/White ethnicity, *n* (%)	11 (47.8)	23 (44.2)	822 (44.3)
Very high HDI country, *n* (%)	15 (65.2)	26 (50.0)	1071 (57.8)
Married/cohabiting, *n* (%)	21 (91.3)	47 (90.4)	1185 (63.9)
Graduate education and above, *n* (%)	19 (82.6)	39 (75.0)	1294 (69.8)
Income: living comfortably, *n* (%)	16 (69.6)	20 (38.5)	690 (37.2)
B. Disease-related factors			
Disease subtype: SAIRD, *n* (%)	13 (56.5)	31 (59.6)	1260 (68.0)
Disease duration, years, median (IQR)	8.0 (4.0–10.0)	7.0 (3.0–12.2)	7.0 (3.0–15.0)
Active disease, *n* (%)	2 (8.7)	18 (34.6)	780 (42.1)
Complex multimorbidity, *n* (%)	0 (0.0)	2 (3.8)	138 (7.4)
Basic multimorbidity, *n* (%)	2 (8.7)	7 (13.5)	469 (25.3)
Mental health comorbidity, *n* (%)	4 (17.4)	16 (30.8)	671 (36.2)
C. Medications			
csDMARDs, *n* (%)	3 (13.0)	16 (30.8)	908 (49.0)
b/tsDMARDs, *n* (%)	7 (30.4)	12 (23.1)	478 (25.8)
Corticosteroids >10 mg/day, *n* (%)	0 (0.0)	3 (5.8)	212 (11.4)
D. Lifestyle factors			
Regular exercise, *n* (%)	11 (47.8)	25 (48.1)	1018 (54.9)
Alcohol use ever, *n* (%)	9 (39.1)	22 (42.3)	926 (49.9)
Current smoker, *n* (%)	1 (4.3)	2 (3.8)	149 (8.0)
E. Primary outcome: self-efficacy (SEMCD)			
SEMCD score (1–10), median (IQR)	7.00 (5.83–8.00)	5.92 (4.50–7.37)	5.50 (4.17–7.00)
SE classification, *n* (%)^a^			
Low SE (≤33rd percentile)	4 (17)	20 (38)	663 (36)
Moderate SE (34th–66th percentile)	5 (22)	13 (25)	604 (33)
High SE (≥67th percentile)	14 (61)	19 (37)	587 (32)
F. Patient-reported outcomes			
Pain VAS (0–10), median (IQR)	2.0 (0.0–3.5)	4.0 (3.0–6.2)	5.0 (3.0–7.0)
Fatigue VAS (0–10), median (IQR)	5.0 (2.0–6.5)	6.0 (4.8–8.0)	6.0 (5.0–8.0)
PROMIS PF-4a T score, median (IQR)	57.0 (43.5–57.0)	46.9 (40.2–48.3)	41.9 (36.7–48.3)
PROMIS PF-4a T score <40, *n* (%)	2 (8.7)	13 (25.0)	741 (40.0)
PROMIS GPH T score (0–100), median (IQR)	47.7 (43.6–52.5)	39.8 (34.9–42.3)	37.4 (32.4–42.3)
PROMIS GPH T score <40, *n* (%)	5 (21.7)	29 (55.8)	1207 (65.1)
G. Psychological and social outcomes			
Resilience (mean BRS (1–5)), median (IQR)	3.3 (2.8–3.9)	3.0 (2.5–3.8)	3.0 (2.5–3.7)
Loneliness scale (3–9), median (IQR)	4.0 (3.0–5.5)	6.0 (3.0–6.0)	6.0 (4.0–7.0)
Family APGAR (0–10), median (IQR)	9.0 (7.5–10.0)	8.0 (5.0–10.0)	8.0 (5.0–10.0)

a Percentages were calculated within each pregnancy/breastfeeding status column. Multimorbidity was defined as the presence of two or more chronic conditions in the same individual. Basic multimorbidity: ≥2 chronic non-rheumatic conditions; complex multimorbidity: ≥3 chronic non-rheumatic conditions affecting ≥3 distinct organ systems, reflecting a greater and more varied comorbidity burden. APGAR: Adaptation, Partnership, Growth, Affection and Resolve; BRS: Brief Resilience Scale; b/tsDMARDs: biologic/targeted synthetic disease-modifying antirheumatic drugs; Continuous variables: median (IQR); csDMARDs: conventional synthetic disease-modifying antirheumatic drugs; HDI: Human Development Index; IQR: interquartile range; PB: pregnant or breastfeeding; PF-4a: Physical Function 4a; PROMIS GPH: Global Physical Health; SAIRD: systemic autoimmune/inflammatory RMD; SE: self-efficacy; SEMCD: Self-Efficacy for Managing Chronic Disease; VAS: visual analogue scale.

SAIRD: systemic autoimmune/inflammatory rheumatic and musculoskeletal diseases, including idiopathic inflammatory myopathies; SLE: Sjögren’s disease, SSc, APS, discoid lupus, MCTD, UCTD, familial Mediterranean fever, IgG4-related disease, PMR, sarcoidosis, Takayasu’s arteritis, GCA, Behcet’s disease, vasculitis and familial Mediterranean fever; IA: inflammatory arthritis, including RA, JIA, Ankylosing spondylitis or axial spondyloarthropathy, enteropathic arthritis, psoriatic arthritis, reactive arthritis.

To our knowledge, this is the first study to investigate the role of SE among pregnant and breastfeeding women living with RMDs. In this small study, pregnant women with RMDs reported high levels of SE in managing their condition, along with lower pain and fatigue and better physical function and physical health. These findings suggest that higher SE may be associated with better patient-reported well-being during pregnancy among individuals with RMDs. However, the results should be interpreted with caution given the small sample size and predominance of participants from high-income countries. Pregnant women with RMD in this sample did not use high-dose corticosteroids and may have had greater access to specialized rheumatology and obstetric care and better-controlled disease, compared with breastfeeding women. Given the cross-sectional design, we cannot establish the direction of the association between SE and disease activity. Larger longitudinal studies are needed to confirm these findings, and our group will continue pursuing this work.

## Supplementary Material

rkag132_Supplementary_Data

## Data Availability

The data that support the findings of this study are available from the corresponding author upon reasonable request.
