# Supplementary material for: Self-efficacy among pregnant and breastfeeding women with rheumatic and musculoskeletal diseases: an international cross-sectional pilot study
Source: Rheumatol Adv Pract. 2026 Oct 24;10(4):rkag132. doi: 10.1093/rap/rkag132 (PMC13744344; doi:10.1093/rap/rkag132)
Supplement: Supplementary file 1 [file rkag132_supplementary_data.docx]

**Supplementary Data S1**

**Self-Efficacy Among Pregnant and Breastfeeding Women with Rheumatic and Musculoskeletal Diseases: An International Cross-Sectional Pilot Study**

**COVAD-3: Study Group Authors and Affiliations**

1. **Algeria**
   a. **Dr Chafia Dahou Makhloufi** - Professor in rheumatology, faculty of medicine. Bab El Oued University Hospital, Algiers, Algeria. ORCID: 0000-0002-6523-9851.
2. **Argentina**
   a. **Dr Leandro Gabriel Ferreyra Garrott** - Rheumatologist. Sección de Reumatologia, Servicio de Clínica Médica. Hospital Italiano de Buenos Aires. ORCID: 0000-0003-3326-7628.
3. **Armenia**
   a. **Dr Valentina Vardanyan** - Yerevan State Medical University, Mikayelyan University Hospital, Yerevan, Armenia. ORCID: 0000-0002-8582-7837.
4. **Australia**
   a. **Dr Vidya Sadanand Limaye** - 1. Rheumatology Department, Royal Adelaide Hospital, Port Rd, Adelaide SA 5000, Australia; 2. Discipline of Medicine, Adelaide University, North Tce, Adelaide SA 5000, Australia. ORCID: 0000-0002-8142-9532.
   b. **Dr Jessica Day** - 1. Department of Rheumatology, Royal Melbourne Hospital, Parkville, VIC 3050, Australia; 2. Walter and Eliza Hall Institute of Medical Research, Parkville, VIC 3052 Australia; 3. Department of Medical Biology, University of Melbourne, Parkville, VIC 3052 Australia. ORCID: 0000-0001-8528-4361.
5. **Bangladesh**
   a. **Dr A T M Tanveer Hasan** - Associate Professor of Rheumatology, Green Life Medical College, Dhaka, Bangladesh. ORCID: 0000-0002-5332-3319.
6. **Belarus**
   a. **Dr Marharyta Volkava** - Belarusian Medical Academy of Post-Graduate Education, Minsk, Republic of Belarus. ORCID: 0000-0001-8572-9252.
7. **Bhutan**
   a. **Dr Sonam Yangchen** - Jigme Dorji Wangchuk National Referral Hospital.
8. **Brazil**
   a. **Dr Dimitri Luz Felipe da Silva -** University Santo Amaro; Hospital Israelita Albert Einstein. ORCID: 0000-0003-0869-9330.
   b. **Dr Odirlei Andre Monticielo -** Division of Rheumatology, Department of Internal Medicine, Hospital de Clínicas de Porto Alegre, Universidade Federal Do Rio Grande Do Sul, Porto Alegre, Brazil. ORCID: 0000-0003-0720-2097.
   c. **Dr Samuel Katsuyuki Shinjo -** Division of Rheumatology, Faculdade de Medicina FMUSP, Universidade de São Paulo, São Paulo, SP, Brazil. ORCID: 0000-0002-3682-4517.
   d. **Dr Simone Appenzeller -** Department of Orthopedics, Rheumatology and Traumatology, School of Medical Science, Universidade Estadual de Campinas, Cidade Universitária, Campinas SP, CEP 13083-970, Brazil. ORCID 0000-0001-5075-4474.
9. **Bulgaria**
   a. **Dr Russka Shumnalieva** - Department of Rheumatology, Clinic of Rheumatology, Medical University-Sofia, Bulgaria. ORCID: 0000-0003-2321-6536.
   b. **Dr Rositsa Svetoslavova Dacheva** - Diagnostic and Consultation Center UMHAT "St.Ivan Rilski", Sofia, Bulgaria. ORCID: 0000-0002-5474-4131.
   c. **Dr Tsvetelina Velikova** - Medical Faculty, St. Kliment Ohridski University, Sofia, Bulgaria. ORCID: 0000-0002-0593-1272.
10. **Cambodia**
    a. **Dr Koy Rattanak** - Rheumatologist, Sonlak joint experts, Phnom Penh, Cambodia.
11. **Cameroon**
    a. **Dr Fernando Kemta Lekpa** - 1. Department of Internal Medicine and Specialties, Faculty of Medicine and Pharmaceutical Sciences, University of Dschang, Dschang, Cameroon; 2. Department of Internal Medicine, Douala General Hospital, Douala, Cameroon. ORCID: 0000-0001-7592-5049.
12. **Canada**
    a. **Dr Océane Landon-Cardinal -** Division of Rheumatology, Centre hospitalier de l'Université de Montréal (CHUM); CHUM Research Center; Department of Medicine, Université de Montréal, Montréal, Québec, Canada. ORCID: 0000-0002-3361-6165.
    b. **Dr Vanessa Ocampo** - Scarborough health network. Kensington eye Institute. University of Toronto. ORCID: 0000-0002-5705-1111.
13. **Chile**
    a. **Dr María Francisca Bozán Pérez** - Universidad de Chile/Hospital Clínico Universidad de Chile. ORCID: [0000-0002-6109-1773](https://orcid.org/0000-0002-6109-1773).
    b. **Dr Antonia Valenzuela** - Depto. de Reumatología e Inmunología Clínica, División de Medicina Interna, Pontificia Universidad Católica de Chile. ORCID: 0000-0003-3357-9402.
14. **China**
    a. **Dr Guochun Wang** - Department of Rheumatology, China-Japan Friendship Hospital, Yinghua East Road, Chaoyang District, Beijing, 100029, China. ORCID: 0000-0002-4616-9376.
15. **Hong Kong**
    a. **Dr Ho So** - The Chinese University of Hong Kong. ORCID: 0000-0001-7113-9390.
    b. **Dr Iris Tang** - Clinical Assistant Professor from the Division of Rheumatology and Clinical Immunology at the Department of Medicine at the University of Hong Kong, Special administrative regions of China. ORCID: 0000-0002-1636-5384.
    c. **Dr Edmund KM Wong** - Department of Medicine and Therapeutics, Prince of Wales Hospital, Hong Kong, Special administrative regions of China. ORCID: 0009-0008-5628-4184.
16. **Colombia**
    a. **Dr Carlos Enrique Toro Gutiérrez** - General Director, Reference Center for Osteoporosis, Rheumatology and Dermatology, Pontifica Universidad Javeriana Cali, Colombia. ORCID: 0000-0002-6084-7049.
    b. **Dr Sebastian Herrera Uribe** - Clínica Las Américas Auna – ARTMÉDICA. ORCID: 0000-0001-7247-1846.
    c. **Dr Carlo Vinicio Caballero-Uribe** - Universidad de la Costa (CUC), Barranquilla, Colombia. ORCID: 0000-0002-9845-8620.
17. **Croatia**
    a. **Dr Ivan Padjen** - Division of Clinical Immunology and Rheumatology, Department of Internal Medicine, University Hospital Centre Zagreb, University of Zagreb School of Medicine. ORCID: 0000-0002-9249-9325.
    b. **Dr Zeljka Kardum** - Department of Rheumatology, Clinical Immunology and Allergology, University Hospital Centre Osijek, J. Huttlera 4, 31000, Osijek, Croatia. School of Medicine, University J. J. Strossmayer Osijek, Huttlera 4, 31000, Osijek, Croatia. ORCID: 0000-0002-6220-3685.
18. **Cyprus**
    a. **Dr Konstantinos Parperis** - University of Cyprus Medical School. ORCID: 0000-0001-6009-0130.
19. **Denmark**
    a. **Dr Karen Schreiber** - 1. Danish Hospital for Rheumatic Diseases, University of Southern Denmark, Sønderborg, Denmark; 2. Institute of Regional Health Research (IRS), University of Southern Denmark, Odense, Denmark; 3. Thrombosis and Haemophilia, Kings Healthcare Partners, Guy's and St Thomas' NHS Foundation Trust, London, UK. ORCID: 0000-0003-0660-7690.
20. **Dominican Republic**
    a. **Dr. Jossiell Then Báez** - MD, Hospital Metropolitano de Santiago (HOMS), Santiago, Dominican Republic. ORCID: 0000-0002-8727-2855.
21. **Egypt**
    a. **Dr Reem Hamdy Abdellatif Mohammed** - Department of Rheumatology and Clinical Immunology and Rehabilitation School of Medicine Cairo University. ORCID: 0000-0003-4994-7687.
    b. **Dr Hala Mohamed Lotfy** - Department of Pediatrics and pediatric rheumatology, Cairo University. ORCID: 0000-0002-2090-925X.
    c. **Dr Walaa Abdel Rahman Saleh** - Rheumatology Department, Faculty of Medicine, Cairo University, Egypt. ORCID: 0000-0002-6531-2692.
    d. **Dr Mervat Eissa** - Rheumatology Department, Kasr Alainy Medical School, Cairo University. ORCID: 0000-0001-7999-3401.
    e. **Dr Tamer A. Gheita** - Rheumatology Department, Faculty of Medicine, Cairo University, Egypt. ORCID: 0000-0002-1155-9729.
22. **Equador**
    a. **Dr Carlos Rios** - Ecuadorian Society of Rheumatology, Guayaquil, Ecuador. ORCID: 0000-0003-0221-8276.
23. **Estonia**
    a. **Dr Sandra Meisalu** - East-Tallinn Central Hospital, Tallinn, Estonia. ORCID: [0000-0001-9307-089X](https://orcid.org/0000-0001-9307-089X).
24. **Ethiopia**
    a. **Dr Becky Abdissa Adugna** - Rheumatology Unit, Internal Medicine Department, Addis Ababa University, Addis Ababa, Ethiopia. ORCID: 0000-0002-9740-4516.
25. **France**
    a. **Dr Margherita Giannini** - Service de Physiologie et Explorations Fonctionnelles Musculaires, University Hospital of Strasbourg; Referral Centre for Rare Autoimmune Diseases, Strasbourg; UR3072, University of Strasbourg, France. ORCID: 0000-0002-4834-5804.
    b. **Dr Julien Campagne -** Internal Medicine Department, University of Lorraine, 34, Cours Leopold, CS 25233, CEDEX, 54052 Nancy, France. ^2^Internal Medicine Department, University Hospital of Nancy, Rue du Morvan, CEDEX, 54511 Vandœuvre-Lès-Nancy, France. ORCID: 0000-0002-6186-4334.
    c. **Dr Alain Meyer** **-** Explorations fonctionnelles musculaires, Centre de Référence des maladies autoimmunes rares, Hôpitaux Universitaires de Strasbourg; UR 3072 Centre de Recherche en biomedecine, Université de Strasbourg; European referrence network ReCONNET.
    d. **Dr Francis Guillemin** - 1. EA 4360 Apemac, Université de Lorraine, Nancy, France. 2. Inserm, CHRU Nancy, CIC-1433 Epidémiologie Clinique, Université de Lorraine, Nancy, France. ORCID: 0000-0002-9860-7024.
26. **Germany**
    a. **Dr Hannah Labinsky** - Department of Internal Medicine II, Rheumatology/Clinical Immunology, University of Würzburg, Wuerzburg, Germany. ORCID: 0000-0001-5762-9182.
    b. **Dr Gerd-Rüdiger Burmester** - Department of Rheumatology and Clinical Immunology, Charité - University Medicine Berlin, Free University and Humboldt University Berlin, Germany. ORCID: 0000-0001-7518-1131.
    c. **Dr Johannes Knitza** - 1. Medizinische Klinik 3 - Rheumatologie und Immunologie, Universitätsklinikum Erlangen, Friedrich-Alexander-Universität Erlangen-Nürnberg, Ulmenweg 18, 91054, Erlangen, Deutschland; 2. Institute of Digital Medicine, University Hospital of Giessen and Marburg, Philipps-University Marburg, Marburg, Germany. ORCID: 0000-0001-9695-0657.
27. **Ghana**
    a. **Dr Dzifa Dey** - Rheumatology Unit, Department of Medicine and Therapeutics, University of Ghana Medical School, College of Health Sciences, Korle-Bu, Accra, Ghana. ORCID: 0000-0002-3339-5112.
28. **Greece**
    a. **Dr Alexandros Panagiotopoulos** - Rheumatology Unit, First Department of Propaedeutic Internal Medicine, Joint Academic Rheumatology Program, Laiko Hospital, Medical School, National & Kapodistrian University of Athens, 11527 Athens, Greece. ORCID: 0000-0003-3612-9394.
    b. **Dr Eirini Vasarmidi** - Department of Respiratory Medicine, University of Crete, School of Medicine, 71500 Crete, Greece.
    c. **Dr Katerina Antoniou** - Laboratory of Cellular and Molecular Pneumonology, School of Medicine, University of Crete, Heraklion, Crete, Greece.
29. **Guatemala**
    a. **Dr Andrea Bran Ordóñez** - Hospital Universitario Esperanza, Hospital El Pilar, PANLAR, AMIG. ORCID: 0000-0002-8758-7021.
30. **Hungary**
    a. **Dr Melinda Nagy-Vincze** - Division of Clinical Immunology, Faculty of Medicine, University of Debrecen, Móricz Zsigmond út 22, Debrecen, H-4032, Hungary. ORCID: [0000-0003-0316-3828](https://orcid.org/0000-0003-0316-3828).
31. **India**
    a. **Dr Vikas Agarwal** - Department of Clinical Immunology and Rheumatology, Sanjay Gandhi Postgraduate Institute of Medical Sciences, Lucknow, India. ORCID: 0000-0002-4508-1233.
    b. **Dr Bhupen Barman** - Additional Professor and Head, Department of Medicine, All India Institute of Medical Sciences (AIIMS), Guwahati. ORCID: 0000-0002-5433-1310.
    c. **Dr Yogesh Preet Singh** - Assistant Professor, Division of Rheumatology and Clinical Immunology, Department of General Medicine, Himalayan Institute of Medical sciences, Swami Rama University, Jolly Grant, Dehradun, Uttarakhand, India. ORCID: 0000-0003-1258-0041.
    d. **Dr Arunkumar R Pande -** Consultant Endocrinologist, Lucknow Endocrine Diabetes and thyroid clinic, India. ORCID: 0000-0002-9570-1509.
    e. **Dr Jasmine Parihar** - Assistant Professor of Neurology, All India Institute of Medical Science, New Delhi, and National Cancer Institute, Jhajjar, India. ORCID: 0000-0002-5545-4304.
    f. **Dr Lekshmi M.R.:** Department of Clinical Immunology and Rheumatology, Sanjay Gandhi Postgraduate Institute of Medical Sciences, Lucknow, India. ORCID: 0009-0003-5921-3684.
    g. **Dr Meera Shah** - Department of Rheumatology, Topiwala National Medical College and B.Y.L Nair Hospital, Mumbai, India. ORCID: 0009-0005-3308-7231.
32. **Indonesia**
    a. **Dr Suryo Anggoro Kusumo Wibowo** - Division of Rheumatology, Department of Internal Medicine, Cipto Mangunkusumo Hospital/Faculty of Medicine, Universitas Indonesia. ORCID: [0000-0002-0089-0504](about:blank).
33. **Iraq**
    a. **Dr Maryam Masoumi** - Clinical research and development center. ORCID: 0000-0003-2635-2656.
    b. **Dr Avin Maroof** - University of Kurdistan Hawlêr. ORCID: 0000-0002-6554-5314.
    c. **Dr Asal Adnan Redha** - MD, Rheumatology Unit, Department of Medicine, Baghdad Teaching Hospital, Medical City, Baghdad, Iraq. ORCID: 0000-0001-9140-5860.
34. **Israel**
    a. **Dr Or Aharonov** - Department of Gerontology, Faculty of Social Welfare and Health Science, University of Haifa, Haifa, Israel. ORCID: 0000-0003-4107-9045.
35. **Italy**
    a. **Dr Marta Mosca** - Rheumatology Unit, University of Pisa, Pisa, Italy. ORCID: 0000-0001-5937-4574.
    b. **Dr. Nicoletta Del Papa** - Unità operativa complessa (UOC) Reumatologia Clinica, ASST G. Pini-CTO, Milano, Italy. ORCID: 0000-0003-1549-8852.
    c. **Dr. Fabiola** **Atzeni** - Rheumatology Unit, University of Messina, Messina, Italy. ORCID: Unavailable.
    d. **Dr Marco Sebastiani** - University of Parma. ORCID: 0000-0002-1294-6421.
    e. **Dr Franco Franceschini** - Department of Clinical and experimental sciences - University of Brescia; Rheumatology and Clinical Immunology Unit - ASST Spedali Civili of Brescia, Italy. ORCID: 0000-0003-3678-6124.
    f. **Dr Pier Paolo Sainaghi** - Department of Translational Medicine, Università del Piemonte Orientale UPO, Novara, Italy; Division of Internal Medicine, Rheumatology Unit, CAAD (Center for Translational Research on Autoimmune and Allergic Disease) Maggiore della Carità, Italy. ORCID: 0000-0001-8322-9158.
    g. **Dr Maria Giovanna Danielli** - Clinica Medica, Dipartimento di Scienze Cliniche e Molecolari, Università Politecnica delle Marche, via Tronto 10A, 60126 Ancona, Italy. ORCID: 0000-0002-9608-2252.
    h. **Dr Vincenzo Venerito** - Rheumatology Unit, Department of Precision and Regenerative Medicine-Ionian Area, University of Bari “Aldo Moro”, Bari, Italy. ORCID: 0000-0002-2573-5930.
    i. **Dr Alessandro Giollo** - Rheumatology Unit, University of Padua, Italy. ORCID: 0000-0001-9355-7673.
    j. **Dr Laura Andreoli** - 1. Rheumatology and Clinical Immunology Unit, ASST Spedali Civili and University of Brescia, 25123 Brescia, Italy; 2. Department of Clinical and Experimental Sciences, University of Brescia, 25123 Brescia, Italy; 3. Danish Centre for Expertise in Rheumatology (CeViG), Danish Hospital for Rheumatic Diseases, Sønderborg, Denmark; 4. Department of Regional Health Research, University of Southern Denmark, Odense, Denmark. ORCID: [0000-0002-9107-3218](https://orcid.org/0000-0002-9107-3218).
    k. **Dr Alessia Alluno** - University of L’Aquila – Department of Life, Health & Environmental Sciences; Internal Medicine and Nephrology Division, ASL 1 Avezzano-Sulmona-L'Aquila, San Salvatore Hospital, L'Aquila, Italy. ORCID: 0000-0003-1105-5640.
    l. **Dr Devis Benfaremo** - Department of Clinical and Molecular Sciences, Marche Polytechnic University, Ancona, Italy. ORCID: 0000-0002-9867-2360.
    m. **Dr Valentino Paci** - Medical Clinic and Internal Medicine Residency Programme, Department of Clinical and Molecular Sciences, Marche Polytechnic University & Department of Internal Medicine, Azienda Ospedaliero-Universitaria delle Marche, Italy. ORCID: [0000-0002-9466-8460](https://orcid.org/0000-0002-9466-8460).
    n. **Dr Giovanni Damiani** - 1. Department of Biomedical, Surgical and Dental Sciences, University of Milan, Milan, Italy; 2. Italian Center of Precisione Medicine and Chronic Inflammation, University of Milan, Milan, Italy. ORCID: 0000-0002-2390-6505.
    o. **Dr Elvis Hysa** - Laboratory of Experimental Rheumatology and Academic Division of Clinical Rheumatology, Department of Internal Medicine, University of Genoa, Italy - IRCCS San Martino Polyclinic, Genoa, Italy. ORCID: [0000-0002-6970-0983](https://orcid.org/0000-0002-6970-0983).
    p. **Dr Matteo Lucchini** - Fondazione Policlinico Universitario Agostino Gemelli IRCCS, Rome Italy; Università Cattolica del Sacro Cuore, Rome, Italy.
    q. **Dr Alberto Lo Gullo** - Unit of Rheumatology, Arnas Garibaldi Catania. ORCID: 0000-0003-4383-0314.
    r. **Dr Lorenzo Cavagna** - Rheumatology Unit, Dipartimento di Medicine Interna e Terapia Medica, Università degli studi di Pavia, Pavia, Lombardy, Italy. ORCID: 0000-0003-3292-1528.
    s. **Dr Marco Fornaro** - University of Bari, Department of Precision and Regenerative Medicine and Ionian Area, Rheumatology Unit, Bari, Italy. ORCID: 0000-0003-1716-7432.
36. **Japan**
    a. **Dr Akira Yoshida** - Department of Allergy and Rheumatology, Nippon Medical School Graduate School of Medicine. ORCID: 0000-0003-3590-1637.
    b. **Dr Saori Abe** - University of Tsukuba. ORCID: 0000-0003-2793-5589.
    c. **Dr Masataka Kuwana** - Department of Allergy and Rheumatology, Nippon Medical School Graduate School of Medicine, 1-1-5 Sendagi, Bunkyo-ku, Tokyo 113-8602, Japan. ORCID: 0000-0001-8352-6136.
    d. **Dr Ran Nakashima (Sasai)** - Department of Rheumatology and Clinical Immunology, Graduate School of Medicine, Kyoto University, 54 Shogoin-Kawahara-cho, Sakyo-ku, Kyoto 606-8507, Japan.
37. **Jordan**
    a. **Dr Fatima Alnaimat** - University of Jordan. ORCID: 0000-0002-5574-2939..
38. **Kazakhstan**
    a. **Dr Gulzhan Trimova** - MD, PhD, Department of Clinical Subjects, al-Farabi Kazakh National Medical University, Almaty, Kazakhstan. City Rheumatology Center, Almaty, Kazakhstan. ORCID: 0000-0001-8130-4150.
    b. **Dr Dana Bekaryssova** - South Kazakhstan Medical Academy: Shymkent, KZ. ORCID: 0000-0002-9651-7295.
    c. **Dr Akerke Auanassova -**Department of Biology and Biochemistry, South Kazakhstan Medical Academy, Shymkent, Kazakhstan. ORCID: 0000-0002-3952-1650.
39. **Kenya**
    a. **Dr Eugene Kalman Genga** - Lecturer at University of Nairobi. ORCID: 0000-0001-5866-7105.
40. **Lebanon**
    a. **Dr Nelly Ziade** - 1. Rheumatology Department, Saint-Joseph University, Beirut, Lebanon; 2. Rheumatology Department, Hotel-Dieu de France Hospital, Beirut, Lebanon. ORCID: 0000-0002-4479-7678.
41. **Libya**
    a. **Dr Soad Salem Hashad** - Tripoli children hospital /university of Tripoli. ORCID: 0000-0003-0500-7458.
    b. **Dr Laila Ayoub** - 1. Faculty of Medicine, University of Tripoli, Rheumatology, Tripoli, Libya; 2. Tripoli Central Hospital, Internal medicine, Tripoli, Libya. ORCID: [0000-0003-0381-2197](about:blank).
42. **Malaysia**
    a. **Dr Syahrul Sazliyana Shaharir** - Department of Medicine, Faculty of Medicine, Universiti Kebangsaan Malaysia. ORCID: 0000-0002-9068-8114.
    b. **Dr Tan Chou Luan** - 1. Rheumatology Unit, Department of Medicine, Hospital Sultanah Bahiyah, Malaysia; 2. Rheumatology Unit, Department of Medicine, Hospital Putrajaya, Malaysia. ORCID: 0000-0002-9859-5385.
43. **Malta**
    a. **Dr Rosalie** **Magro** - 1. Rheumatology Department, Mater Dei Hospital, Tal-Qroqq, Msida, MSD, 2090, Malta; 2. Faculty of Medicine and Surgery, University of Malta, Msida, Malta. ORCID: 0000-0001-8486-8410.
44. **Mexico**
    a. **Dr Erick Adrian Zamora Tehozol** - Centro Médico Pensiones, Mexico. ORCID: [0000-0002-7888-3961](https://orcid.org/0000-0002-7888-3961).
    b. **Dr Ignacio García-De La Torre -** Universidad de Guadalajara. Department of Immunology and Rheumatology, Hospital General de Occidente. Guadalajara, Jal. México. ORCID: 0000-0002-9261-678X.
    c. **Dr Iris J. Colunga‑Pedraza** - Hospital Universitario UANL monterrey Mexico, Mexico. ORCID: 0000-0002-2786-5843.
    d. **Dr** **Javier Merayo-Chalico** - Reumátika, Centro de Vanguardia en Reumatología, Mexico City. ORCID: 0000-0002-5870-0523.
    e. **Dr Deshire Alpizar-Rodriguez** - Research Unit, Mexican College of Rheumatology. ORCID: 0000-0002-6930-0517.
    f. **Dr Miguel Angel Saavedra Salinas** - Research Division, Hospital de Especialidades Dr. Antonio Fraga Mouret, CMN La Raza, IMSS, Mexico City. ORCID: 0000-0003-0687-9944.
    g. **Dr Abraham Edgar Gracia-Ramos** - Department of Internal Medicine, General Hospital, National Medical Center La Raza, Instituto Mexicano del Seguro Social, Av. Jacaranda S/N, Col. La Raza, Alc. Azcapotzalco, C.P. 02990 Mexico City, Mexico. ORCID: 0000-0003-1842-2554.
45. **Moldova**
    a. **Dr Victoria Sadovici-Bobeica** - State University of Medicine and Pharmacy “Nicolae Testemitanu”. ORCID: 0000-0003-1803-6960.
46. **Mongolia**
    a. **Dr Zulgerel Dandii** - Mongolian national University of Medical sciences.
47. **Morocco**
    a. **Dr Ihsane Hmamouchi -** 1. Health Sciences Research Centre (CReSS), Morocco; 2. Faculty of Medicine, International University of Rabat (UIR), Morocco. ORCID: 0000-0003-4402-5034.
48. **Nepal**
    a. **Dr Binit Vaidya** - Department of Rheumatology, National Centre for Rheumatic Diseases, Ratopul, Kathmandu, Nepal. ORCID: [0000-0002-4840-8924](https://orcid.org/0000-0002-4840-8924).
49. **Netherlands**
    a. **Dr Luis Fernando Perez Garcia** - Department of Rheumatology, Erasmus Medical Center, Rotterdam, Netherlands. ORCID: 0000-0002-8958-9493.
50. **New Zealand**
    a. **Dr Nicola Dalbeth** - Department of Medicine, University of Auckland, Auckland, New Zealand. ORCID: 0000-0003-4632-4476.
51. **Nicaragua**
    a. **Dr** **Idania Calixta Escalante Mendoza** - Oscar Danilo Rosales Arguello School Hospital. ORCID: 0000-0003-0156-8098.
    b. **Dr Christian Mauriel Uriarte Hernandez** - Hospital Vivian Pellas. Clínica de Reumatología. ORCID: 0009-0009-3424-7961.
52. **Niger**
    a. **Dr Moussa Elh Ibrahim Fanata** - Head of the Rheumatology Department, Niamey National Hospital, Niger.
53. **Nigeria**
    a. **Dr Uyiekpen Ima-Edomwonyi -** Consultant, Department of Internal Medicine, Lagos University Teaching Hospital, Lagos, Nigeria. ORCID: 0000-0001-7651-5025.
    b. **Dr Ibukunoluwa Dedeke** - Consultant Rheumatologist and Physician, University College Hospital Ibadan, Oyo state Nigeria; Associate lecturer, College of Medicine, University of Ibadan. ORCID: [0000-0002-6494-0654](https://orcid.org/0000-0002-6494-0654).
    c. **Dr Airenakho Emorinken** - Rheumatology Division, Department of Internal Medicine, Irrua specialist Teaching Hospital, Irrua, Edo State. ORCID: [0000-0002-3306-572X](https://orcid.org/0000-0002-3306-572X).
    d. **Dr Henry Madu Nwankwo** - Department of internal medicine, Faculty of Medicine, Nnamdi Azikiwe University, Awka, Nnewi Campus Anambra State Nigeria. ORCID: [0000-0002-5679-7093](https://orcid.org/0000-0002-5679-7093).
    e. **Dr Hakeem Babatunde Olaosebikan** - Consultant Rheumatologist, Lagos State University College of Medicine/Lagos State University Teaching Hospital, Ikeja, Lagos, Nigeria. +2348035751154. ORCID: 0000-0003-4004-0093.
    f. **Dr Celestine Okwara**: Rheumatology, University of Nigeria Teaching Hospital, Enugu, Nigeria. ORCID: 0000-0002-7727-5657.
    g. **Dr Tralagba Uchechukwu** - University of Port Harcourt Teaching Hospital, Port Harcourt, Rivers State, Nigeria.
54. **Oman**
    a. **Dr Batool Hassan** - Dr Kowther Hassan Dr Talal Al Lawati. ORCID: 0000-0002-5954-0189.
55. **Pakistan**
    a. **Dr Uzma Rasheed** - Pakistan Institute of Medical Sciences Islamabad, Shaheed Zulfiqar Ali Bhutto Medical University. ORCID: 0009-0001-5122-0703.
    b. **Dr Babur Salim -** Rheumatology Department, Fauji Foundation Hospital, Rawalpindi, Pakistan. ORCID: 0000-0001-8430-9299.
56. **Paraguay**
    a. **Dr Nelly Colman Mc Leod** - Universidad Nacional de Asunción, Hospital de Clínicas de la Facultad de Ciencias Médicas, Departamento de Reumatología. Asunción, Paraguay. ORCID: 0000-0001-8709-6567.
57. **Peru**
    a. **Dr Manuel Francisco Ugarte-Gil** - Grupo Peruano de Estudio de Enfermedades Autoinmunes Sistémicas, Universidad Cientifica del Sur, Lima, Peru & Rheumatology Department, Hospital Nacional Guillermo Almenara Irigoyen, EsSalud, Lima, Peru. ORCID: 0000-0003-1728-1999.
    b. **Dr Victor Pimentel-Quiroz** - Hospital Nacional Guillermo Almenara Irigoyen/Universidad Científica del Sur. ORCID: 0000-0002-3638-7054.
58. **Philippines**
    a. **Dr Lisa S Traboco -** 1. St Luke's Medical Center-Global City; 2. University of the Philippines-Manila, MIU. ORCID: 0000-0002-1952-7879.
59. **Poland**
    a. **Dr Joanna Makowska** - Department of Rheumatology, Medical University of Lodz. ORCID: 0000-0003-2036-375X.
    b. **Dr Aleksandra Opinc-Rosiak** - Department of Rheumatology, Medical University of Lodz, Zeromskiego 113, 90-549 Lodz, Poland. ORCID: 0000-0002-7881-5731.
    c. **Dr Marcin Milchert** - Department of Internal Medicine, Rheumatology, Diabethology, Geriatrics and Clinical Immunology of Pomeranian Medical University in Szczecin. ORCID: 0000-0002-0943-8768.
60. **Portugal**
    a. **Dr Luís Sousa Inês** - 1. Rheumatology Department, Centro Hospitalar Universitário de Coimbra EPE, Coimbra, Portugal; 2. Faculty of Health Sciences, Universidade da Beira Interior. Covilhã, Portugal. ORCID: 0000-0003-3172-3570.
    b. **Eduardo Dourado -** 1. Rheumatology Department, Unidade Local de Saúde da Região de Aveiro, Aveiro, Portugal; 2. Aveiro Rheumatology Research Centre, Egas Moniz Health Alliance, Aveiro, Portugal. Rheumatology Research Unit, Instituto de Medicina Molecular, Faculdade de Medicina, Universidade de Lisboa, Lisboa, Portugal.
61. **Qatar**
    a. **Dr Samar Al Emadi** - Medicine Department, Rheumatology Section, Hamad Medical Corporation, Doha, Qatar. ORCID: 0000-0001-7942-4831.
62. **Romania**
    a. **Dr Cristina Alexandru** - Internal Medicine and Rheumatology Department, Dr. Ion Cantacuzino Clinical Hospital, 011437 Bucharest, Romania. ORCID: 0000-0001-7053-7530.
    b. **Dr Anca Bobircă** - 1. Internal Medicine and Rheumatology Department, Dr. Ion Cantacuzino Clinical Hospital, 011437 Bucharest, Romania; 2. Department of Rheumatology and Internal Medicine, Carol Davila University of Medicine and Pharmacy, 050474 Bucharest, Romania. ORCID: 0000-0002-6662-7354.
    c. **Dr** **Claudia Cobilinschi** - 1. Department of Rheumatology, Sfânta Maria Clinical Hospital, 011172, Bucharest, Romania; 2. Department of Rheumatology and Internal Medicine, Carol Davila University of Medicine and Pharmacy, 050474 Bucharest, Romania. ORCID: 0000-0002-9117-2187.
63. **Russian Federation**
    a. **Dr Margarita Aleksandrovna Gromova** - Pirogov Russian National Research Medical University (RNRMU), Moscow, Russian Federation. ORCID: 0000-0002-3757-058X.
64. **Rwanda**
    a. **Dr Mubirigi Alexandre:** Pediatrician, Kacyiru Police Hospital, Kigali, Rwanda. ORCID: 0000-0003-3413-9855.
65. **Saudi Arabia**
    a. **Dr Lina El Kibbe** - Division of Rheumatology, Department of Internal Medicine, Specialized Medical Center, Riyadh, Saudi Arabia. ORCID: 0000-0003-1710-9996.
    b. **Dr Hussein Mohammed Halabi** - Department of medicine, section of rheumatology, King Faisal specialist hospital and research center, Jeddah, Saudi Arabia. ORCID: 0000-0002-5174-8292.
66. **Serbia**
    a. **Dr Rada Miskovic** - Clinic of Allergy and Immunology, University Clinical Centre of Serbia; Faculty of Medicine, University of Belgrade. ORCID: 0000-0003-0982-2572.
67. **Singapore**
    a. **Dr Anindita Santosa** - Division of Rheumatology, Aaria Rheumatology, Gleneagles Hospital, Singapore. ORCID: 0000-0002-7320-4552.
    b. **Dr Tze Chin Tan** - Department of Rheumatology and Immunology and Department of Internal Medicine, Singapore General Hospital.
    c. **Dr Desmond Chua** - Tan Tock Seng Hospital, Singapore.
68. **Spain**
    a. **Dr Cristiana Sieiro Santos** - Rheumatology Department, Complejo Asistencial Universitario de León, León, Spain. ORCID: 0000-0003-0889-9877.
    b. **Dr Jesús Loarce-Martos** - Ramón y Cajal University Hospital, Madrid, Spain. ORCID: 0000-0003-1352-9539.
    c. **Dr Sergio Prieto-González** - Department of Internal Medicine and Autoimmune Diseases. Hospital Clínic of Barcelona. IDIBAPS. University of Barcelona, Spain. ORCID: 0000-0003-3179-597X.
    d. **Dr Juan Molina-Collada** - Hospital General Universitario Gregorio Marañón, Madrid, Spain. ORCID: 0000-0001-5191-7802.
69. **Sri Lanka**
    a. **Dr Chathurika Lakmini Dandeniya** - Senior Lecturer at the Department of Medicine, Faculty of Medicine, University of Peradeniya and Honorary Consultant Rheumatologist at the University Teaching Hospital, Peradeniya. ORCID: 0000-0001-8857-1366.
70. **Sudan**
    a. **Dr Ahmed Seri Ibrahim Mohamed** - Clinical Immunology and Allergy center, Royal Care International Hospital. ORCID: 0000-0002-6516-5450.
    b. **Dr Manasik Mamoun -** Faculty of Medicine, Al Neelain University, Sudan.ORCiD: 0009-0009-7303-3018.
71. **Sweden**
    a. **Dr Ioannis Parodis** - 1. Division of Rheumatology, Department of Medicine Solna, Karolinska Institutet, Karolinska University Hospital, and Center for Molecular Medicine (CMM), Stockholm, Sweden; 2. Department of Rheumatology, Faculty of Medicine and Health, Örebro University, Örebro, Sweden. ORCID: 0000-0002-4875-5395.
72. **Switzerland**
    a. **Dr Raphael Micheroli** - Department of Rheumatology, University Hospital Zurich, University of Zurich, Zurich, Switzerland. ORCID: [0000-0002-8918-7304](https://orcid.org/0000-0002-8918-7304).
    b. **Dr Lisa Christ** - Department of Rheumatology and Immunology, Inselspital, Bern University Hospital, University of Bern, Bern, Switzerland. ORCID: 0000-0002-7358-2051.
    c. **Dr Oliver Distler** - Department of Rheumatology, University Hospital Zurich, University of Zurich, Switzerland. ORCID: 0000-0002-0546-8310.
73. **Syria**
    a. **Dr Nada Alchama** - Consultant rheumatologist in Syria.IbN alnafis hospital. ORCID: 0000-0001-6305-4581.
74. **Taiwan**
    a. **Dr Yi-Ming Chen** - Division of Allergy, Immunology, and Rheumatology, Department of Internal Medicine, Taichung Veterans General Hospital, Taichung, Taiwan; Department of Post-Baccalaureate Medicine, College of Medicine, National Chung Hsing University, Taichung, Taiwan. ORCID: 0000-0001-7593-3065.
    b. **Dr Chih-Wei Tseng** - 1. Division of Allergy, Immunology and Rheumatology, Department of Internal Medicine, Taichung Veterans General Hospital, Taichung, Taiwan; 2. Department of Public Health, College of Medicine, National Cheng Kung University, Tainan, Taiwan. ORCID: 0000-0002-5948-7306.
75. **Thailand**
    a. **Dr Phonpen Akarawatcharangura** - Department of Medicine, Queen Savang Vadhana Memorial Hospital, Chonburi, Thailand. ORCID: [0009-0009-7611-9992](https://orcid.org/0009-0009-7611-9992)**.**
    b. **Dr Wanruchada Katchamart** - Division of Rheumatology, Department of Medicine, Faculty of Medicine Siriraj Hospital, Mahidol University, Bangkok, Thailand. ORCID 0000-0002-8952-5967.
    c. **Dr Kotchakorn Kaewboot** - Division of Rheumatology, Department of Medicine. Faculty of Medicine Siriraj Hospital, Mahidol University. Bangkok, Thailand.
    d. **Dr Pichaya O-charoen** - Division of Allergy Immunology and Rheumatology. Faculty of Medicine Ramathibodi Hospital Mahidol University. Bangkok, Thailand.
76. **Tunisia**
    a. **Dr Wafa Hamdi** - Department of Rheumatology Kassab Ksar Said Institute Tunis Tunisia- Faculty of medecine of Tunis - Tunis El Manar University. ORCID: 0000-0003-3045-4831.
77. **Turkey**
    a. **Dr Döndü Üsküdar Cansu** - Prof.Dr (Division of Rheumatology, Department of Internal Medicine,Eskişehir Osmangazi University School of Medicine: Eskişehir, TR). ORCID: 0000-0001-6543-3905.
    b. **Dr Reşit Yıldırım** - Osmangazi University, Division of Rheumatology. ORCID: 0000-0003-4040-0212.
    c. **Dr Tugba Izci Duran** - Denizli State Hospital. ORCID: 0000-0003-4428-9873.
    d. **Dr Neslihan Gokcen** - Kocaeli university faculty of medicine. ORCID: 0000-0003-3022-493X.
78. **Ukraine**
    a. **Dr** **Myroslava Kulyk** - Bogomolets National Medical University, Internal Medicine Department No 2, Kyiv, Ukraine. ORCID: 0000-0002-7695-9977.
79. **United Arab Emirates**
    a. **Dr Nesreen Mohamed Ismail Moustafa** - Rheumatology Department- Suez Canal University, Ismailia, Egypt. ORCID: 0000-0002-4900-1675.
80. **United Kingdom**
    a. **Dr Latika Gupta** - 1. Department of Rheumatology, Royal Wolverhampton Hospitals NHS Trust, Wolverhampton, United Kingdom; 2. Department of Inflammation and Ageing, School of Infection, Inflammation and Immunology, University of Birmingham; 3. Francis Crick Institute, London, United Kingdom. ORCID: 0000-0003-2753-2990.
    b. **Dr Elena Nikiphorou** - 1. Centre for Rheumatic Diseases, Faculty of Life Sciences and Medicine, King's College London, London, UK. 2. Rheumatology Department, King's College Hospital NHS Foundation Trust, London, UK. 3. Centre for Education, Faculty of Life Sciences and Medicine, King’s College London, London, UK. ORCID: 0000-0001-6847-3726.
    c. **Dr John D Pauling** - 1. Royal National Hospital for Rheumatic Diseases (at Royal United Hospitals), Upper Borough Walls, Bath, BA1 1RL, UK. 2. Department of Pharmacy and Pharmacology, University of Bath, Bath, UK. ORCID: [0000-0002-2793-2364](https://orcid.org/0000-0002-2793-2364)**.**
    d. **Dr Chris Wincup** - Department of Rheumatology, King’s College Hospital, London, United Kingdom. ORCID: 0000-0002-8742-8311.
    e. **Dr Ai Lyn Tan** - 1. NIHR Leeds Biomedical Research Centre, Leeds Teaching Hospitals Trust, Leeds, UK; 2. Leeds Institute of Rheumatic and Musculoskeletal Medicine, University of Leeds, Leeds, UK. ORCID: 0000-0002-9158-7243.
    f. **Dr Tania Gudu** - 1. Rheumatology Department, Ipswich Hospital, ESNEFT, Ipswich, UK 2- Psychology Department, EEPRU, University of Essex, Colchester, UK. ORCID: 0000-0002-8973-323X.
    g. **Dr Valentina Pucino** - Institute of Inflammation and Ageing (Birmingham), Kennedy Institute of Rheumatology (Oxford). ORCID: 0000-0002-7683-4568.
    h. **Dr Sarah Dyball** - 1. Centre for Epidemiology Versus Arthritis, Division of Musculoskeletal and Dermatological Sciences, Faculty of Biology, Medicine and Health, University of Manchester, Manchester Academic Health Science Centre, Manchester, UK; 2. Kellgren Centre for Rheumatology, Manchester University Hospitals NHS Foundation Trust, Manchester, UK. ORCID: 0000-0001-6201-7391.
    i. **Dr James Lilleker** - 1. Centre for Musculoskeletal Research, Division of Musculoskeletal and Dermatological Sciences, School of Biological Sciences, Faculty of Biology, Medicine and Health, Manchester Academic Health Science Centre, The University of Manchester, Manchester, UK; 2. Neurology, Manchester Centre for Clinical Neurosciences, Northern Care Alliance NHS Foundation Trust, Salford, UK. ORCID: 0000-0002-9230-4137.
    j. **Dr Aurelie Najm** - School of Infection and Immunity, College of Medical Veterinary and Life Sciences, University of Glasgow, Glasgow, UK. ORCID: 0000-0002-6008-503X.
    k. **Dr** **Arvind Nune** - Southport and Ormskirk Hospital NHS Trust, Southport, PR8 6PN, UK. ORCID: 0000-0002-3849-614X.
    l. **Dr Yusuf A. Rajabally -** Honary Professor of Neurology, Aston Medical School, Aston University, Birmingham, UK. ORCID:0000-0002-7170-8343.
    m. **Dr Anne-Marie Russell** - 1. School of Medicine and Health, University of Birmingham, Edgbaston, Birmingham, UK; 2. School of Health and Care Professions, University of Exeter, Exeter, Devon, UK; 3. ILD Regional Service, University Hospitals Birmingham NHS Trust, Birmingham, UK.
81. **United States of America**
    a. **Dr Aarat Patel** - Bon Secours Rheumatology Center and Division of Pediatric Rheumatology, Department of Pediatrics, University of Virginia, Bon Secours Mercy Health, USA. ORCID: 0000-0002-9756-2274.
    b. **Dr Shikha Singla** - Medical College of Wisconsin. ORCID: 0000-0002-5540-8850.
    c. **Dr Tulika Chatterjee** - University of Illinois College of Medicine at Peoria, Illinois. ORCID: 0000-0001-8844-851X.
    d. **Dr Ashima Makol** - Division of Rheumatology, Mayo Clinic, Rochester, MN, USA. ORCID: 0000-0002-8748-898X.
    e. **Dr. Mazen Dimachkie:** Department of Neurology, SOM-Kansas City Associate Director, Associate Director of the Institute for Neurological Discoveries, SOM-Kansas City, Neurology Clinical Director, Director, Neuromuscular Division, SOM-Kansas City, Neurology. ORCID: 0000-0002-7148-989X.
    f. **Dr Vahed Maroufy** - Department of Biostatistics and Data Science, School of Public Health, The University of Texas Health Center at Houston, Austin, TX 77002, United States. ORCID: 0000-0002-6653-7586.
82. **Venezuela**
    a. **Dr Yurilís Fuentes-Silva** - Departamento de Medicina, Universidad de Oriente, Ciudad Bolívar, Venezuela. ORCID: 0000-0002-5915-769X.
83. **Vietnam**
    a. **Dr Bich Ngoc Nguyen** - Bach Mai Hospital, Hanoi, Vietnam, Karolinska University Hospital.
    b. **Lan Nguyen** - 1. Department of Biochemistry, Hanoi Medical University, Hanoi, Vietnam; 2. Clinical Laboratory, Hanoi Medical University Hospital, Hanoi, Vietnam.
    c. **Hung Nguyen** - Department of Internal Medicine, Hanoi Medical University, Hanoi, Vietnam.
    d. **Thu Hoai Pham** - 1. Hanoi Medical University, Hanoi, 100000, Vietnam; 2. Hanoi Medical University Hospital, Hanoi Medical University, 1 Ton That Tung, Dong Da, Hanoi, 100000, Vietnam.
84. **Zambia**
    a. **Dr Panganani** **Njobvu** - University of Lusaka, School of Medicine and Levy Mwanawasa Teaching hospital, Lusaka, Zambia. ORCID: 0000-0002-1432-0836.
85. **Honduras**
    a. **Dr Hugo Alonzo** - Jefe del Departamento de Medicina Interna en Hospital de Especialidades del Seguro Social en Tegucigalpa, Honduras.
    b. **Dr Carlos Benito Santiago Pastelin** - Médico especialista, Instituto Hondureño del Seguro Social, Honduras.
86. **Panama**
    a. **Dr Generoso Guerra Bautista** - Centro de Investigación Marbella, Paitilla Panamá, Panamá.
    b. **Dr Enrique Julio Giraldo Ho** - Rheumatologist, Universidad de Panamá, República de Panamá.

**Patient Research Partners**

a. **Mr Peter Boyd:** 1. Services Support Officer, Arthritis Ireland; 2. Chair, EULAR PARE Committee. ORCID: 0009-0009-4753-8717.
b. **Ms Linda Kobert:** Research and Communications Specialist, The Myositis Association. ORCID: 0000-0002-3374-1003.
c. **Dr Karen Cheng:** 1. Patient Research Partner/Volunteer, EULAR PARE; 2. MIHRA Foundation, Patient Advisory Board Member, New Orleans, United States of America. ORCID: 0009-0004-3850-8480.
d. **Ms Paula Jordan:** Honorary general secretary, Myositis UK.
e. **Ms Kirtida Oza:** Co-founder Director, Sjogren’s India, Ahmedabad, India.
f. **Ms Ingrid De Groot:** Chair Dutch Myositis Working Group, Patient Association for Neuromuscular Diseases, Baarn, The Netherlands.
g. **Ms Allison Foss:** Executive Director, Myasthenia Gravis Association.
h. **Ms Celia Meyer:** Board Member; Myasthenia Gravia Foundation of America.
i. **Ms Karin Blomkvist Sporre:** Lupus Europe, PAN, Stockholm, Sweden.
j. **Ms Annika Broberg Lavén:** Board Member, National Association for SLE.
k. **Ms Veronica Fatura: Patient Research Partner:** TMA Michigan Support Group Co-leader, TMA Adelante Affinity Group Co-leader.
l. **Ms Ailsa Bosworth:** NRAS.
m. **Ms Malak Aburas:** Arab Adult Arthritis Awareness Group.
n. **Ms Silvia Aguilera:** Vice-President of SAF Espana, ePAG at ERN-ReCONNET, patient representative within the EULAR Study Group on Reproductive Health and Family Planning (ReHFaP). ORCID: 0000-0001-6021-5335.
o. **Ms Rachel Bromley:** The Myositis Association.
